# Gender difference in self-reported empathy: Effects of task instructions and exposure to gender essentialism primes

**DOI:** 10.1371/journal.pone.0337211

**Published:** 2025-12-12

**Authors:** Namitha Rajasekhar, Anna Redly, Shevantika Nanda, Gillian R. Brown

**Affiliations:** School of Psychology & Neuroscience, University of St Andrews, St Andrews, United Kingdom; Public Library of Science, UNITED STATES OF AMERICA

## Abstract

Women often score higher on average than men on self-report measures of empathy. However, self-report estimates of empathic tendencies and other attributes could be susceptible to a range of biases. For instance, participants might respond in a manner that is socially desirable and aligns with gender stereotypes about empathic abilities. We examined whether gender differences in self-reported empathy were affected by a) manipulating task instructions or b) priming with fictive narratives describing gender differences as either fixed or malleable. In Study 1, participants (N = 154) completed questionnaire measures of empathy, social desirability and acceptance of stereotyping. Contrary to our prediction, gender differences in self-reported empathy were not larger when participants were told that we were measuring ‘empathy’. However, in both genders, average scores were higher for empathic concern in the ‘empathy’ condition than in the control condition, which suggests that describing the task as measuring empathy encouraged both male and female participants to present themselves as showing concern for others. Also, participants who scored higher on social desirability scored higher on empathic concern, suggesting a link between motivation to conform to social expectations and self-reported affective empathy. In Study 2, participants (N = 155) completed questionnaire measures of empathy, personality and gender essentialism. Gender differences in self-reported empathy were not larger in the condition that primed gender essentialism. However, women who scored high on empathic concern were more likely to align themselves with feminine adjectives, suggesting a link between self-reported consideration for others and feminine attributes. In both studies, on average, women scored significantly higher than men on self-reported empathic tendencies. Although the experimental manipulations did not impact empathy scores in either study, self-reported empathy appears to be related to social desirability and broader social attitudes, which suggests that a range of cultural and social factors might contribute to gender differences in empathy.

## Introduction

Empathy can be broadly defined as the ability to understand, and share in, the emotional states of others [1]. Three specific processes underlie the construct of empathy, namely i) the ability to recognise and correctly attribute particular emotions to others (cognitive component), ii) susceptibility to sharing the emotional states of others (affective component), and iii) motivation or willingness to improve the emotional state of another individual, for example, by helping to reduce their distress (prosocial concern) [1,2]. According to gender-based stereotypes in Western societies, women are expected to show more orientation towards the wellbeing of others than are men [3–5]. Evolutionary theory has been used to argue that past selection has favoured enhanced emotional and empathic abilities in women because of the benefits derived from close social relationships and the requirement to be sensitive to the needs of offspring [6,7]. From this perspective, gender differences in empathic and prosocial tendencies, and the associated gender stereotypes, are assumed to reflect relatively immutable differences in empathic abilities and motivation between the genders.

An alternative view of gender differences in social abilities and behaviour is provided by *social role theory* [8]. In this framework, gendered divisions of labour are underpinned by physical differences between the genders, including the fact that women carry pregnancies and breastfeed infants, and such divisions of labour are said to be supported by childhood socialisation practices that ready individuals for the activities they are likely to undertake in adulthood [9]. Social role theory asks to what extent observed gender differences in psychological traits and abilities might be underpinned by social and cultural processes rather than reflecting evolved psychological mechanisms in the brain. While men and women are reported to engage different neural pathways by when undertaking empathy-related tasks [10], such neurophysiological evidence does not distinguish between these alternative explanations for how these differences in neural processing might arise. In addition, social cognition and empathic tendencies vary across populations and time periods [11,12], and empathic abilities can be enhanced through learning processes and influenced by early life environments [13,14]. Collectively, these findings suggest that gender differences in empathy are likely to, at least partially, reflect the impact of gendered social norms on developmental processes.

The empirical evidence documenting gender differences in empathy is substantial, with most studies reporting that women and girls exhibit higher empathic tendencies than do men and boys [2,7]. Self-report questionnaires are often used, whereby participants are asked to what extent they agree with statements about their own empathic and prosocial responses. Several self-report measures are available [15,16], including the *Empathy Quotient* [17], which has demonstrated gender differences favouring women in large sample sizes [18]. The *Interpersonal Reactivity Index* [19,20] is another commonly used self-report measure that assesses empathic tendencies by presenting participants with a set of items about perspective-taking during social interactions (PT), empathic concern for others (EC), personal distress in emotional situations (PD), and engagement with fictional material (F). The first two subscales (PT and EC) are thought to represent the cognitive and affective components of empathy respectively [21,22], and inconsistencies are often found in terms of which subscales exhibit significant gender differences [23–25], suggesting some variability and flexibility in empathic tendencies reported by women and men.

One issue with relying on self-report measures of empathic tendencies is that respondents might answer questionnaires in a manner that conforms to social expectations [26]. This *social desirability bias* might lead participants to respond in a socially appropriate manner, guided, for instance, by stereotypes, and thereby over-report socially desirable behaviour and under-report undesirable behaviour. Self-reported estimates of cognitive empathy (e.g., perspective-taking ability) are shown to be only a weak predictor of behavioural empathic skills (e.g., emotion recognition) [27,28], and participants are particularly inaccurate at reporting their own social skills [29]. In terms of gender differences, some tasks that require participants to interpret the emotional states of others exhibit smaller gender differences than do self-report empathy measures [30]; for example, the ‘reading-the-mind-in-the-eyes’ task often shows only small effect sizes favouring women or generates no significant gender differences in task scores [25,31,32]. In addition, monetary rewards have been shown to eliminate gender differences in the accuracy of ascribing emotions to others [33] and to increase participants’ willingness to engage in perspective-taking [34]. Thus, self-reported empathic tendencies might not accurately reflect respondents’ abilities or behaviour when presented with opportunities to exhibit empathy.

These lines of evidence lead to the prediction that self-reported empathic tendencies in participants will be sensitive to the setup of the task or the experimental protocol. To test the effects of making participants aware that they are being evaluated on empathy, thereby activating gender stereotypes, Nanda [35] conducted a pilot study in which male and female participants (N = 20 each) completed the IRI after being explicitly told that this questionnaire measures either ‘empathy’ or ‘social skills’. On average, women scored higher than men on the IRI in the ‘empathy’ condition but not in the ‘social skills’ condition, as predicted if the ‘empathy’ condition elicited gender stereotyping and socially desirable responding, although a recent study failed to replicate this finding using a similar design [36]. An earlier study by Klein and Hodges [33] also failed to find an effect of manipulating task instructions on empathic abilities using an experimental paradigm that involved watching a video of a target who had recently received disappointing news and being asked to infer the feelings of that person at specific timepoints in the video. Another study primed female participants with material that stated, contrary to popular wisdom, women do not have higher emotional intelligence than men [37]: these female participants scored higher, on average, on an emotion recognition task than those in a control condition, suggesting that the women in the experimental condition were motivated to disprove the threat to the traditional view of female superiority on emotion-based tasks. Gender differences in empathy have thus been proposed to vary according to a range of aspects of experimental design, including whether or not the measures are self-report [30,38], but the findings of these previous studies have been inconsistent.

Individuals who endorse social stereotypes (e.g., gender stereotypes) are likely to hold ‘essentialist’ views about human traits (i.e., the belief that members of a category, such as ‘women’ or ‘men’, share fixed, inborn, biologically based attributes or *essences*) [39]. Responses on self-report measures of empathy could thus be related to whether individuals are exposed to, and endorse, gender essentialist views. Clark and colleagues [40] presented participants with a narrative comprising of fictive neurological evidence that either women or men are typically more empathetic. Female participants scored higher, on average, than men on self-reported empathy in the ‘women-better’ condition, but not the ‘men-better’ condition, where the average male score was raised to the female-typical level. These findings suggest that male participants were sensitive to the essentialist information and more likely to describe themselves as empathic when told that this trait was male-typical. Similarly, Pang and colleagues [24] reported that gender differences in self-reported empathy were absent in a condition where women and men were primed with fictive scientific evidence that people of their own gender are most caring about the feelings of others. Learning about the genetic or evolutionary bases of between-group differences, whether fictitious or genuine, has been suggested to increase the likelihood that human traits are perceived as immutable and natural [41,42]. Exposure to essentialist views could thus influence whether individuals perceive themselves as having stereotypically gendered traits.

The aim of our research was to examine the short-term effects of a) manipulating task instructions, and b) priming with gender essentialist narratives, on self-reported empathy across women and men, as well as investigating the links between self-reported empathy and the tendency to respond in a socially desirable manner and endorse stereotypic and essentialist views. In Study 1, participants were administered the IRI, where the IRI task instructions either stated that the questionnaire measured i) ‘empathy’ or ii) ‘social skills’, as in previous studies [35,36]. We predicted that female participants would have higher average IRI scores than male participants in the ‘empathy’ condition and that the gender difference would be smaller, or absent, in the ‘social skills’ condition. Participants in Study 1 also completed a social desirability scale (*Social Desirability Scale*) [43] and a stereotype endorsement scale (*Acceptance of Stereotyping Questionnaire*) [44]. In Study 2, participants were primed with fictitious scientific evidence that ascribed gender differences to either i) biological factors (‘pro-essentialism’ condition) or ii) social processes (‘anti-essentialism’ condition), using primes from a previous study [45]. We predicted that the gender difference in average IRI scores would be present in the pro-essentialism condition and smaller, or absent, in the anti-essentialism condition. Participants in Study 2 also stated the extent to which they aligned with masculine/feminine self-descriptors (*Gender-Stereotypic Characteristics*) [46] and reported their gender essentialist views (*Gender Essentialism Scale*) [47].

## Study 1: Manipulation of task instructions

### Materials and methods

#### Participants.

Participants were recruited via Prolific (www.prolific.co), and, in total, 160 respondents completed the questionnaires. Data from participants who did not provide information about their gender, or selected non-binary/another preferred gender term, were removed (N = 4), given the binary-gender comparative framework of the study, and the data from one female participant were removed, as the IRI was only partially completed. The final sample therefore consisted of 155 participants (77 women, 78 men). Most of the participants were 18–29 years of age (18–29 years = 109 participants; 30–39 years = 33; 40–49 years = 12; 50 + years = 1). All data were collected anonymously, and participants gave their informed consent through selecting the consent option at the start of the online survey. Participants were reimbursed for their time. Recruitment took place between 1^st^ and 10^th^ September 2021, and ethical approval was provided in advance by the School of Psychology & Neuroscience Ethics Committee (University of St Andrews, UK).

#### Measures.

i) *Interpersonal Reactivity Index (IRI)* [19,20] This measure consists of 28 items that ask about various aspects of empathy and concern for others. It consists of four subscales: *Perspective-Taking* (PT; e.g., ‘*I sometimes try to understand my friends better by imagining how things look from their perspective*’), *Empathic Concern* (EC; e.g., ‘*When I see someone being taken advantage of, I feel kind of protective toward them*’), *Personal Distress* (PD; e.g., ‘*In emergency situations, I feel apprehensive and ill-at-ease*’) and *Fantasy* (F; e.g., ‘*When I am reading an interesting story or novel, I imagine how I would feel if the events in the story were happening to me*’), each represented by 7 items. Using a 5-point Likert scale, respondents state how much they agree that each statement describes them (1 = ‘strongly disagree’, 5 = ‘strongly agree’), and nine items require reverse coding. Total IRI scores are calculated by averaging across all responses for each participant, and high scores represent high levels of self-reported empathy. The IRI has demonstrated satisfactory internal consistency and reliability [19] (Cronbach’s α in current study = 0.774).
ii) *Social Desirability Scale* (*SDS)* [43] This 16-item measure consists of statements that assess whether respondents conform to social expectations and avoid breaking social contracts (e.g., ‘*I always accept others’ opinions, even when they don’t agree with my own*’). Although the original scale employed true/false responses, a 5-point Likert scale was used here for consistency with the other measures (1 = ‘strongly disagree’, 5 = ‘strongly agree’); previous research has suggested that Likert-type scales could be better than dichotomous measures for identifying social desirability [48], which supports our use of a Likert scale for this measure. Six items require reverse coding, and responses are combined into a total mean score, where high scores represent high levels of self-reported conformity to social expectations. The scale has demonstrated satisfactory internal consistency and reliability [43] (Cronbach’s α in current study = 0.766). No additional validation or psychometric checks beyond Cronbach’s α were performed, so we remain cautious in interpreting this measure, given the use of a Likert scale in our study.
iii) *Acceptance of Stereotyping Questionnaire (ASQ)* [44] This 12-item measure asks about the extent to which respondents accept the importance or usefulness of stereotyping (e.g., ‘*You cannot get through life without generalizing about people, even though such generalizations may be overstated*’). Due to experimenter error, only 11 items were provided to participants; one item was omitted by mistake (‘*Stereotypes are useful in daily life even though they are not always correct*’), but the Cronbach’s α values were comparable between this study and the original. Respondents select the extent to which they agree with each statement, using a 5-point Likert scale (1 = ‘strongly disagree’, 5 = ‘strongly agree’). Five items require reverse coding, and the responses are combined into a total mean score, where high scores represent stronger acceptance of stereotyping. The scale has demonstrated satisfactory internal consistency and reliability [44] (Cronbach’s α in current study = 0.773).

#### Procedure.

The study was administered to participants via Qualtrics. After providing informed consent, participants were randomly assigned to one of two conditions via random allocation by the survey platform (Qualtrics):

i) the *Empathy* (E) condition, in which the IRI task instructions explicitly stated that the IRI measures *empathic abilities*:

‘This questionnaire asks about your ability to *understand another person’s point of view or feelings and understand the need to show empathy towards another person*. It also asks about your ability to react *with* *an appropriate emotional response after identifying the need to show empathy*’ (italics added).

ii) the *Social Ability* (SA) condition, in which the IRI task instructions omitted the word ‘empathy’ and instead implied that the IRI measures broader *social abilities*:

‘This questionnaire asks about your ability to *decipher another person’s thoughts, identify complex situations and realise the need to take action*. It also asks about your ability to react *in* *an appropriate manner after identifying a situation where action is necessary*’ (italics added).

After being shown the instructions and completing the IRI, participants in both conditions completed the SDS and ASQ in randomised order. Participants were then debriefed and redirected to Prolific for reimbursement.

#### Statistical analyses.

The analyses were conducted in SPSS (v28). All data derived from Likert scales were treated as continuous and are presented as means ± SEMs. As the data conformed to the assumptions of parametric tests (skewness, kurtosis and Shapiro-Wilks’ normality tests on residuals), multivariate ANOVAs were used to explore the main effects and interactions (gender and condition). If significant condition x gender interactions had been detected for IRI scores, further analyses were planned to examine whether SDS or ASQ scores mediated any gendered effects of condition on self-reported empathy; given the lack of significant interactions, no exploratory mediation analyses were conducted. Pearson’s correlations were conducted for pair-wise comparisons between variables by combining the data within gender across conditions and employing a conservative alpha value (≤.005) to account for multiple comparisons.

### Results

#### Gender and condition.

**Fig 1:**
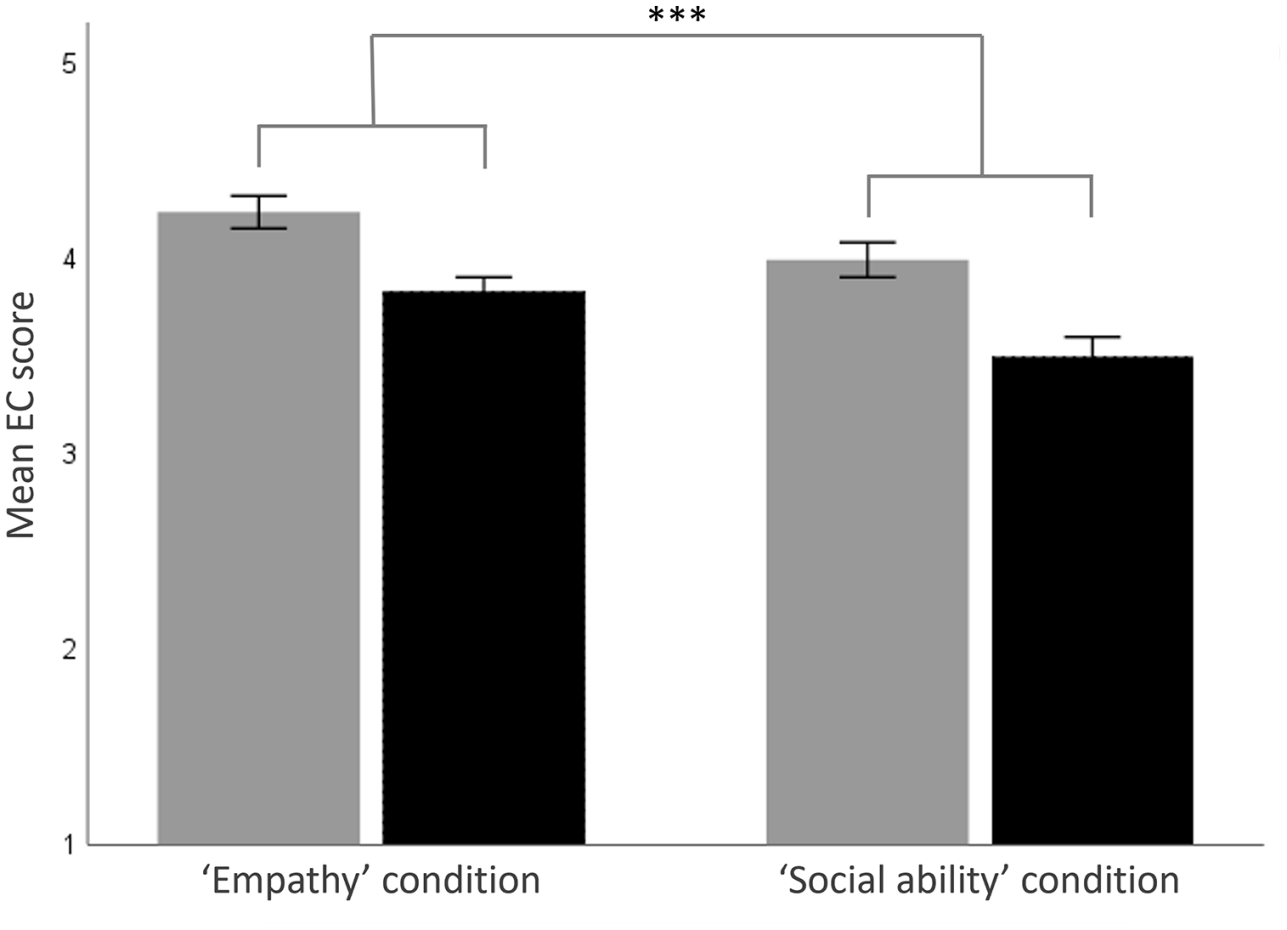
Empathic Concern scores in two experimental conditions in Study 1. Mean (± SEM) score on the Empathic Concern (EC) subscale of the *Interpersonal Reactivity Index* in the *Empathy* condition and *Social Ability* condition in Study 1, where grey bars represent female participants and black bars represent male participants. *** p = 0.001.

On average, women scored higher than men on the total IRI (female participants: 3.75 ± 0.05; male participants: 3.45 ± 0.04; *F*_1,151 _= 24.38, p < 0.001; Table 1); this gender difference was also found for the IRI_EC (*F*_1,151 _= 26.83, p < .001), IRI_PD (*F*_1,151 _= 10.69, p < .001) and IRI_F subscales (*F*_1,151 _= 5.08, p = .026) but was marginally non-significant for the IRI_PT subscale (*F*_1,151 _= 3.84, p = .052). Neither the main effect of condition (*F*_1,151 _= 1.35, p = .247), nor the interaction between gender and condition (*F*_1,151 _= 0.01, p = .944), was significant for the total IRI score. However, main effect of condition was significant for the IRI_EC subscale (*F*_1,151 _= 11.01, p = .001; Fig 1), as a results of participants in the E condition scoring higher, on average, on this subscale than participants in the SA condition. The interaction between gender and condition was not significant for the IRI_EC subscale, and the main effects of condition and interactions between gender and condition were not significant for any of the other IRI subscales (*F* and p values in Table 1). While no gender difference in mean scores was found on the SDS (*F*_1,151 _= 0.08, p = .779), a significant main effect of gender was found for ASQ, with women scoring lower, on average, than men on this scale (female participants: 2.70 ± 0.06; male participants: 2.97 ± 0.07; *F*_1,151 _= 8.76, p = .004; Table 1). The main effects of condition, and interactions between condition and gender, were not significant for scores on the SDS and ASQ (*F* and p values in Table 1).

**Table 1:** Mean scores for men and women in Study 1.

Scale	Condition	Men (mean ± SEM)	Women (mean ± SEM)	Statistics
**IRI_total**	E	3.48 ± 0.06	3.78 ± 0.06	***Gender*: *F***_**1,151 **_= 24.38, p < .001*** Condition: *F*_1,151 _= 1.35, p = .247 *Interaction*: *F*_1,151 _=* *0.01, p = .944
	SA	3.41 ± 0.06	3.72 ± 0.06	
**IRI_PT**	E	3.61 ± 0.09	3.77 ± 0.10	*Gender*: *F*_1,151 _= 3.84, p = .052 *Condition*: *F*_1,151 _= 0.17, p = .677 *Interaction*: *F*_1,151 _= 0.08, p = .774
	SA	3.63 ± 0.10	3.84 ± 0.09	
**IRI_EC**	E	3.83 ± 0.09	4.24 ± 0.09	***Gender*: *F***_**1,151 **_= 26.83, p < .001*** Condition: *F*_**1,151 **_= 11.01, p = .001*** Interaction: *F*_1,151 _= 0.27, p = .605
	SA	3.50 ± 0.09	4.00 ± 0.09	
**IRI_PD**	E	2.86 ± 0.10	3.28 ± 0.11	***Gender*: *F***_**1,151 **_= 10.69, p = .001** Condition: *F*_1,151 _= 0.10, p = .753 *Interaction*: *F*_1,151 _= 0.40, p = .530
	SA	2.97 ± 0.11	3.25 ± 0.11	
**IRI_F**	E	3.62 ± 0.10	3.84 ± 0.11	***Gender*: *F***_**1,151 **_= 5.08, p = .026* Condition: *F*_1,151 _= 0.49, p = .486 *Interaction*: *F*_1,151 _= 0.01, p = .916
	SA	3.54 ± 0.11	3.78 ± 0.10	
**SDS**	E	3.34 ± 0.08	3.37 ± 0.09	*Gender*: *F*_1,151 _= 0.08, p = .779 *Condition*: *F*_1,151 _= 0.16, p = .689 *Interaction*: *F*_1,151 _= 0.01, p = .939
	SA	3.38 ± 0.09	3.40 ± 0.82	
**ASQ**	E	2.89 ± 0.09	2.67 ± 0.10	***Gender*: *F***_**1,151 **_= 8.76, p = .004** Condition: *F*_1,151 _= 1.44, p = .233 *Interaction*: *F*_1,151 _= 0.52, p = .471
	SA	3.07 ± 0.10	2.72 ± 0.09	

Mean (± SEM) scores on the *Interpersonal Reactivity Index* (IRI_total), IRI subscales (IRI_PT: *Perspective-Taking*; IRI_EC: *Empathic Concern*; IRI_PD: *Personal Distress*; IRI_F: *Fantasy*), *Social Desirability Scale* (SDS) and *Acceptance of Stereotyping Questionnaire* (ASQ) for women and men in the *Empathy* (E) and *Social Ability* (SA) conditions in Study 1. * p ≤ .05, ** p ≤ .01, *** p ≤ .001.

#### Correlations between measures.

In female participants, IRI_EC scores were positively correlated with both IRI_PT (*r* = 0.492, p < .001) and IRI_F scores (*r* = 0.397, p < .001; Table 2). SDS scores correlated positively with IRI_PT (*r* = 0.581, p < .001) and IRI_EC scores (*r* = 0.324, p = .004), and negatively with ASQ scores (*r* = −0.377, p < .001), indicating that women who reported conforming to social expectations were more likely to describe themselves as taking the perspectives of others, showing empathic concern and rejecting stereotypes. ASQ scores also correlated negatively with IRI_PT (*r* = −0.349, p = .002), as women who scored high on acceptance of stereotypes were less likely to describe themselves as engaging in perspective-taking. In male participants, IRI_EC scores correlated positively with IRI_PT scores (*r* = 0.336, p = .003; Table 2), and SDS scores correlated positively with IRI_EC scores (*r* = 0.347, p = .002), indicating that men who reported conforming to social expectations were more likely to describe themselves as showing empathic concern.

**Table 2:** Correlations between measures in Study 1.

	Scale	IRI_PT	IRI_EC	IRI_PD	IRI_F	SDS	ASQ
**Women**	**IRI_PT**						
	**IRI_EC**	0.492***					
	**IRI_PD**	−0.143	0.276				
	**IRI_F**	0.217	0.397***	0.274			
	**SDS**	0.581***	0.324**	−0.172	0.139		
	**ASQ**	−0.349**	−0.119	0.060	0.042	−0.377***	
**Men**	**IRI_PT**						
	**IRI_EC**	0.336**					
	**IRI_PD**	−0.164	0.052				
	**IRI_F**	0.152	0.220	0.269			
	**SDS**	0.280	0.347**	−0.165	−0.151		
	**ASQ**	0.032	−0.243	−0.231	−0.094	0.026	

Pearson’s correlation coefficients for the *Interpersonal Reactivity Index* (IRI) subscales (IRI_PT: *Perspective-Taking*; IRI_EC: *Empathic Concern*; IRI_PD: *Personal Distress*; IRI_F: *Fantasy*), *Social Desirability Scale* (SDS) and *Acceptance of Stereotyping Questionnaire* (ASQ) for women and men in Study 1. ** p ≤ .005, *** p ≤ .001.

## Study 2: Exposure to gender essentialism primes

### Materials and methods

#### Participants.

Participants were recruited via Prolific, and 154 respondents completed the questionnaires. Data from participants who did not provide information about their gender, or selected non-binary or another preferred gender term, were removed (N = 2), given the design of the study. The final sample consisted of 152 participants (77 women, 75 men). The majority of participants were 18–39 years of age (18–29 years = 33 participants; 30–39 years = 59; 40–49 years = 30; 50 + years = 30); the slightly older distribution of participants in Study than in Study 1 is purely an artifact of sample availability and open recruitment practices. All data were collected anonymously, and participants gave their informed consent through selecting the consent option at the start of the online survey. Participants were reimbursed for their time. Recruitment took place between 27^th^ June and 6^th^ July 2022, and ethical approval was provided in advance by the School of Psychology & Neuroscience Ethics Committee (University of St Andrews, UK).

#### Measures.

i) *Interpersonal Reactivity Index (IRI)* [19,20] Participants completed the 28-item IRI that was described in Study 1. Total IRI scores are calculated by averaging across all responses. The IRI demonstrated satisfactory internal consistency and reliability (Cronbach’s α in current study = 0.801).
ii) *Gender-Stereotypic Characteristics* (*GSC)* [46] This measure consists of a list of descriptive words that are typically associated with female and male stereotypes in the domains of personality, cognitive and physical traits. In total, 24 *feminine* words (e.g., ‘gentle’, ‘intuitive’ and ‘pretty’) and 24 *masculine* words (e.g., ‘competitive’, ‘analytical’ and ‘strong’) were presented to participants (the original 36 items, plus 12 items from the negative personality list). Using a 5-point Likert scale, respondents were asked the extent to which each characteristic described themselves (1 = ‘does not describe me’, 5 = ‘describes me extremely well’). Although both male and female participants responded to all items, we were specifically interested in levels of conformity to own-gender descriptors, so, i) for female participants, the average score for feminine descriptors was calculated, and ii) for male participants, the average score for masculine descriptors was calculated, with high scores representing high levels of conformity to feminine, or masculine, descriptors respectively. The scale has demonstrated satisfactory internal consistency and reliability [46] (Cronbach’s α in current study = 0.846).
iii) *Gender Essentialism Scale (GES)* [47] This 25-item measure was designed to capture the extent to which respondents endorse essentialist views of gender, in terms of considering differences between genders as discrete, biologically determined, and invariant (e.g., ‘*Differences between men and women in behaviour and personality are largely determined by genetic predisposition*’). Respondents state the extent to which they agree with each statement, using a 5-point Likert scale (1 = ‘strongly disagree’, 5 = ‘strongly agree’). Five items require reverse coding, and the responses are combined into a total mean score, with high scores representing strong endorsement of gender essentialist views. The scale has demonstrated satisfactory internal consistency and reliability [47] (Cronbach’s α in current study = 0.932).

#### Procedure.

The study was administered to participants via Qualtrics. After providing informed consent, participants were randomly assigned to one of two conditions via random allocation by the survey platform (Qualtrics):

i) the *Pro-Essentialism* (PE) condition, in which participants were provided with a fictious media article, based on a previous study [45] (for the full primes, see the supplementary material in this previous study) describing the supposed genetic underpinnings of differences between the genders, including disease susceptibility:

[Shortened excerpt] ‘Recent research… has confirmed that cells within a human body are gendered… because male cells have an X and a Y chromosome, and female cells have two X chromosomes… All difference between men and women stem from this difference… [M]uch of who we are as people can be traced to our genetic origins – including our gender.’

ii) the *Anti-Essentialism* (AE) condition, in which participants were provided with a fictious media article, based on a previous study [45], describing the supposed social underpinnings of differences between the genders, including disease susceptibility:

[Shortened excerpt] ‘Recent research… has confirmed that men and women are 99.9% genetically similar… Despite the fact that male cells have an X and a Y chromosome, and female cells have two X chromosomes… [T]he environment must play a larger role… [M]uch of who we are as people can be traced to our environment rather than our genetics – including our gender.’

As in the original study [45], the primes were followed by a short set of comprehension questions (5 per condition) to encourage participants to engage with the priming material. The answers to these comprehension questions were coded as correct/incorrect, and the total number of correct answers was calculated for each participant (range = 0–5). After being shown the instructions and completing the IRI, participants in both conditions completed the GSC and GES in randomised order. Participants were then debriefed and redirected to Prolific for reimbursement.

#### Statistical analyses.

The analyses were conducted in SPSS (v28). All data derived from Likert scales were treated as continuous and are presented as means ± SEMs. As the data conformed to the assumptions of parametric tests (skewness, kurtosis and Shapiro-Wilks’ normality tests on residuals), multivariate ANOVAs were used to explore the main effects and interactions (gender and condition). If significant condition x gender interactions had been detected for IRI scores, further analyses were planned to examine whether GSC or GES scores mediated any gendered effects of condition on self-reported empathy. Pearson’s correlations were conducted for pair-wise comparisons between variables by combining the data within gender across conditions and employing a more conservative alpha value (≤.005) to account for multiple comparisons. The number of correct responses to the comprehension questions were compared to chance levels using one-sample *t*-tests.

### Results

#### Comprehension questions.

In both conditions, participants scored significantly higher than chance for the comprehension questions that followed the priming material (PE condition: mean score ± SEM = 4.66 ± 0.10, *t*_76_ = 30.42, p ≤ .001; AE condition: mean score ± SEM = 4.76 ± 0.08, *t*_78_ = 39.71, p ≤ .001). No cut-off was applied, and data from all participants were included in the subsequent analyses.

#### Gender and condition.

As in Study 1, women scored higher, on average, than men on the total IRI (female participants: 3.55 ± 0.04; male participants: 3.23 ± 0.04; *F*_1,148 _= 33.44, p < .001; Table 3); this gender difference was also found for all of the subscales (IRI_PT: *F*_1,148 _= 8.21, p = .005; IRI_EC: *F*_1,148 _= 12.51, p < .001; IRI_PD: *F*_1,148 _= 6.15, p = .014; IRI_F: *F*_1,148 _= 13.59, p < .001). Neither the main effect of condition (*F*_1,148 _= 0.34, p = .854), nor the interaction between gender and condition (*F*_1,148 _= 0.37, p = .542), was significant for the total IRI score. Similarly, none of the main effects of condition, or interactions between gender and condition, were significant for the IRI subscales (*F* and p values in Table 3). No significant gender differences were found for mean scores on the GSC (*F*_1,148 _= 3.17, p = .077) or GES (*F*_1,148 _= 2.90, p = .091; Table 3). The main effects of condition, and interactions between condition and gender, were also not significant for total scores on the GSC and GES (*F* and p values in Table 3).

**Table 3:** Mean scores for men and women in Study 2.

Scale	Condition	Women (mean ± SEM)	Men (mean ± SEM)	Statistics
**IRI_total**	PE	3.54 ± 0.06	3.25 ± 0.05	***Gender*: *F***_**1,148 **_= 33.44, p < .001*** Condition: *F*_1,148 _= 0.34, p = .854 *Interaction*: *F*_1,148 _=* *0.37, p = .542
	AE	3.56 ± 0.05	3.21 ± 0.06	
**IRI_PT**	PE	3.68 ± 0.11	3.45 ± 0.08	***Gender*: *F***_**1,148 **_= 8.21, p = .005** Condition: *F*_1,148 _= 0.10, p = .758 *Interaction*: *F*_1,148 _= 0.01, p = .916
	AE	3.72 ± 0.08	3.46 ± 0.08	
**IRI_EC**	PE	3.97 ± 0.10	3.66 ± 0.09	***Gender*: *F***_**1,148 **_= 12.51, p < .001*** Condition: *F*_1,148 _= 0.43, p = .512 *Interaction*: *F*_1,148 _= 0.23, p = .635
	AE	3.95 ± 0.09	3.54 ± 0.12	
**IRI_PD**	PE	2.96 ± 0.12	2.71 ± 0.09	***Gender*: *F***_**1,148 **_= 6.15, p = .014* Condition: *F*_1,148 _= 0.93, p = .336 *Interaction*: *F*_1,148 _= 0.07, p.793
	AE	2.88 ± 0.11	2.58 ± 0.12	
**IRI_F**	PE	3.53 ± 0.11	3.19 ± 0.10	***Gender*: *F***_**1,148 **_= 13.59, p < .001*** Condition: *F*_1,148 _= 0.996, p = .320 *Interaction*: *F*_1,148 _= 0.219, p = .640
	AE	3.68 ± 0.09	3.25 ± 0.12	
**GSC**	PE	2.40 ± 0.08	2.41 ± 0.10	*Gender*: *F*_1,148 _= 3.17, p = .077 *Condition*: *F*_1,148 _= 0.23, p = .632 *Interaction*: *F*_1,148 _= 3.50, p = .063
	AE	2.61 ± 0.07	2.29 ± 0.10	
**GES**	PE	2.80 ± 0.11	3.05 ± 0.09	*Gender*: *F*_1,148 _= 2.90, p = .091 *Condition*: *F*_1,148 _= 1.85, p = .175 *Interaction*: *F*_1,148 _= 0.48, p = .486
	AE	2.73 ± 0.10	2.83 ± 0.12	

Mean (± SEM) scores on the *Interpersonal Reactivity Index* (IRI_total), IRI subscales (IRI_PT: *Perspective-Taking*; IRI_EC: *Empathic Concern*; IRI_PD: *Personal Distress*; IRI_F: *Fantasy*), *Gender-Stereotypic Characteristics* (GSC) and *Gender Essentialism Scale* (GES) for women and men in the *Pro-Essentialism* (PE) and *Anti-Essentialism* (AE) conditions in Study 2. * p ≤ .05, ** p ≤ .01, *** p ≤ .001.

#### Correlations between measures.

In female participants, IRI_EC scores were positively correlated with IRI_PT scores (*r* = 0.485, p < .001; Table 4). IRI_EC scores also correlated positively with GSC scores (*r* = 0.419, p < .001), indicating that women who reported showing empathic concern were more likely to describe themselves using feminine terms. In male participants, IRI_EC scores were positively correlated with IRI_PT scores (*r* = 0.488, p < .001). In addition, GES scores correlated negatively with IRI_F scores in male participants (*r* = −0.353, p = .002), indicating that men who endorsed gender essentialist views reported lower emotional engagement in fictional material than men who less strongly endorsed gender essentialism.

**Table 4:** Correlations between measures in Study 2.

	Scale	IRI_PT	IRI_EC	IRI_PD	IRI_F	GSC	GES
**Women**	**IRI_PT**						
	**IRI_EC**	0.485***					
	**IRI_PD**	−0.158	−0.083				
	**IRI_F**	0.117	0.048	−0.043			
	**GSC**	0.175	0.419***	−0.113	0.130		
	**GES**	0.050	0.083	0.187	0.156	0.106	
**Men**	**IRI_PT**						
	**IRI_EC**	0.488***					
	**IRI_PD**	−0.192	−0.046				
	**IRI_F**	0.143	0.277	−0.208			
	**GSC**	−0.135	−0.212	−0.234	0.009		
	**GES**	−0.214	−0.236	0.054	−0.353**	0.210	

Pearson’s correlation coefficients for the *Interpersonal Reactivity Index* (IRI) subscales (IRI_PT: *Perspective-Taking*; IRI_EC: *Empathic Concern*; IRI_PD: *Personal Distress*; IRI_F: *Fantasy*), *Gender-Stereotypic Characteristics* (GSC) and *Gender Essentialism Scale* (GES) for women and men in Study 1. ** p ≤ .005, *** p ≤ .001.

## Discussion

The aim of this research was to examine whether gender differences in self-reported empathy were sensitive to task instructions and priming material that aimed to elicit socially desirable responding in line with gender stereotypes. In both studies, women scored significantly higher, on average, than men on self-reported empathic tendencies, as measured by total interpersonal reactivity index (IRI) scores. Yet, contrary to our predictions, neither task instructions nor the priming material influenced this gender difference in self-reported empathy. In Study 1, scores for empathic concern (IRI_EC) were higher on average for participants in the ‘empathy’ condition, which suggests that describing the task as measuring empathy provoked both male and female participants to present themselves as showing concern for others. Both men and women who scored high on social desirability (SDS) were likely to score high on empathic concern, which suggests a link between motivation to conform to social expectations and self-reported affective components of empathy. In Study 2, women who scored high on empathic concern were more likely to align with feminine adjectives in the gender-stereotypic characteristics (GSC) scale, supporting a link between feminine attributes and self-reported consideration for others. These findings add to the existing literature by suggesting that, while the wording of task instructions for empathy measures only has marginal impacts on how participants respond on measures of self-reported empathy, scores on these measures are more robustly related to gender, levels of social desirability and, in women, extent of alignment with traditional gender norms. In addition, men who scored high on gender essentialism (GES) reported lower emotional engagement with fictional material (e.g., books and movies; IRI_F), suggesting a link between essentialist views of gender and depth of emotional responding. Thus, collectively, these data indicate that gender differences in self-reported empathy appear to be susceptible to social desirability biases, and between-individual differences appear to be related to broader social attitudes regarding gender roles.

While significant gender differences in total IRI scores have been found in previous research [23–25], whether gender differences are also found for each of the IRI subscales has shown inconsistencies between studies. For instance, Pang and colleagues [24] found a significant difference only for the personal distress subscale, whereas Baez and colleagues [23] reported significant gender differences for all subscales in a similar sample size. Here, women scored higher, on average, than men on the empathic concern, personal distress and fantasy subscales of the IRI, but not the perspective-taking subscale, in Study 1, while, in Study 2, the gender difference was significant for all IRI subscales. Potential differences in the robustness of gender differences across the subscales of the IRI deserves further investigation. In Study 1, women scored lower, on average, than men on acceptance of stereotypes (ASQ), as reported previously [40], but no gender differences were found for social desirability scores (SDS), consistent with previous research [43]. In Study 2, no differences were found in the average scores of men and women on either the gender-stereotypic characteristics scale (GSC), which indicates that both genders were similar in ascribing gender-typical adjectives to themselves, or the gender essentialism scale (GES). Men are more likely than women to endorse gender essentialism in some countries (e.g., Australia) [47] but not others (e.g., Denmark; UK: this study) [47], which is perhaps related to whether or not men believe that their higher status is being threatened by social change [49]. Future studies could further investigate which factors influence patterns of gender differences in essentialist beliefs, including cross-cultural effects.

Task instructions were manipulated in Study 1, and we predicted that the gender difference in self-reported empathy would be larger in the ‘empathy’ condition than in the ‘social ability’ condition, based on the assumption that the ‘empathy’ instructions would elicit socially desirable responding in line with the gender stereotype that women are more empathic than men. However, the interaction between condition and gender was not significant for the total IRI score or any of the IRI subscales. The lack of interaction effects could have resulted from participants failing to engage with the instruction material or could perhaps have resulted from both sets of instructions eliciting stereotyped ideas about gender differences in emotional and social skills. Despite pilot research suggesting that these primes would influence IRI scores [35], subsequent research indicated that this effect is not consistently found [36]. While the main effect of condition was also non-significant for total IRI scores in the current research, participants in the ‘empathy’ condition scoring higher, on average, than participants in the ‘social ability’ condition on the EC subscale, which suggests that the ‘empathy’ task instructions encouraged participants of both genders to describe themselves as showing concern for others. Therefore, future studies could continue to examine the potential effects of manipulating task instructions on the subscales of self-reported empathy using primes that more explicitly target empathy-related stereotypes, such as those used previously [40], with additional considerations of sample sizes and the diversity of participant pools (e.g., with respect age, ethnicity and socioeconomic background).

In Study 2, priming participants with material that either emphasised pro-essentialist or anti-essentialist views of gender differences did not influence average scores on the IRI or any of its subscales. Previous research has suggested that gender differences in self-reported empathy are sensitive to fictive evidence that either men or women have better empathic skills [24,40], with the findings appearing to show that men’s empathy scores are particularly likely to differ across conditions and be higher when told that men have superior empathic abilities. The priming material in the current study referred to gender differences in general, including disease susceptibility, rather than focusing on empathy or emotional skills, which might have resulted in the null effect. Therefore, priming material that relates broadly to gender essentialism, without specifically mentioning gender stereotypes about empathy, might be unlikely to influence self-reported empathic tendencies, and future studies could potentially use essentialist priming material that is more directly related to empathic abilities. Alternatively, both texts might have primed participants to be sensitive to gender essentialism. Yet, in Study 2, the comprehension questions confirmed that participants engaged with the priming material, and exposure to the specific anti-essentialism prime used in this study has been associated with greater support for women’s rights [45], which suggests that this methodology is worth pursuing. As essentialist primes convey the view that gender differences are immutable [42], future studies could continue to explore whether exposure to essentialist views influence whether individuals perceive themselves as having stereotypically gendered traits across a range of characteristics, including by using relevant priming designs.

Showing empathy is generally considered to be a desirable trait [50], as it can lead to positive consequences for the wellbeing of others. Previous studies have suggested that individual differences in social desirability are correlated with self-reported empathy [26,51]. In line with this research, in Study 1, both men and women who reported conforming to socially desirable expectations were more likely to describe themselves as exhibiting empathic concern, and women who scored high on social desirability were also more likely to describe themselves as engaging in perspective-taking. Thus, self-reported empathy potentially relates to an individual’s commitment to a positive self-description and possibly also to impression management, as well as their moral standpoint, and self-reported empathy therefore might not accurately predict the performance of empathy in behavioural tasks in all instances. These findings might not be specific to self-reported empathy, given that social desirability is also associated with overclaiming in other domains [52]. With regard to moral positioning, women who scored low on acceptance of stereotyping were more likely to score high on social desirability and describe themselves as engaging in perspective-taking, which suggest that, in women, self-reported empathy broadly relates to thinking about others’ perspectives and being open to a diversity of viewpoints, which means that social desirability is potentially a confounder of gender differences in empathy. Given that complex relationships exist between empathy and morality [53], future studies could potentially investigate whether such relationships differ between men and women.

Gender-related traits have been suggested to follow a continuum rather than a dichotomous distribution [54], and empathic responses might therefore reflect the extent to which individuals identify with traditional feminine or masculine descriptors. In Study 2, women who identified with feminine adjectives were more likely to score high on self-reported empathic concern than were women who scored low on this measure. This finding is consistent with the stereotyped notion that concern for others is associated with feminine characteristics, while no correlations between empathy measures and masculinity scores were found for male participants in Study 2. Self-reported empathy has been linked with moral responding, such that individuals with feminine traits are more likely to view social moral conflicts (e.g., conflicts between considering the welfare of others versus upholding rights or duties) as being important and to score higher on prosocial personality traits [55]. Thus, gender differences in empathy are potentially related to broader attitudes to interpersonal relationships. Another finding from Study 2 also suggests a link between depth of emotional responding and social attitudes. Men who scored higher on endorsement of gender essentialist views reported lower emotional engagement with fictional material (as measured by the IRI_F), raising the possibility that emotional components of empathy, in particular, might relate to broader social beliefs such as gender essentialism. Exposure to literary fiction has been associated with lower essentialism [56], and reading literary fiction is thought to improve theory of mind [57], suggesting that empathic tendencies might be sensitive to early life educational experiences and learned beliefs about the fixedness of human psychological traits.

In summary, although the current study reported that, on average, women scored higher than men on a self-report measure of empathy, this gender difference is potentially underpinned by numerous factors, including social desirability biases, the role of gender stereotypes and broader social attitudes. The experimental manipulation of task instructions, and exposure to priming material that promoted essentialist views of gender differences, did not influence self-reported empathy. While these findings might reflect the specific wording of the instructions and priming material used in our studies, issues with priming designs have led to many inconsistent findings in the wider literature [58], which suggests that such designs are unlikely to produce robust effects in many instances. Despite the lack of priming effects on gender differences in self-reported empathy, our results indicated that the ‘empathy’ task instructions did encourage participants of both genders to describe themselves as showing empathetic concern. Also, in both men and women, conformity to socially desirable expectations was related to empathic concern, and women who scored low on acceptance of stereotyping were more likely to describe themselves as engaging in perspective-taking. These findings collectively suggest a link between social attitudes and self-reported empathic tendencies. Social role theory would suggest that socialisation effects influence psychological traits in adulthood, and gender differences in self-reported empathy might be strongly influenced by such factors.
